# Cyberattack on Flight Safety: Detection and Mitigation Using LoRa

**DOI:** 10.3390/s21134610

**Published:** 2021-07-05

**Authors:** Rony Ronen, Boaz Ben-Moshe

**Affiliations:** Department of Computer Science, Ariel University, Ariel 40700, Israel; rony.ronen@msmail.ariel.ac.il

**Keywords:** ADS-B, cyber, Internet of Things (IoT), LoRa, sense and avoid, Wireless Sensor Networks (WSNs)

## Abstract

Automatic Dependent Surveillance-Broadcast (ADS-B) is the main communication system currently being used in Air Traffic Control (ATC) around the world. The ADS-B system is planned to be a key component of the Federal Aviation Administration (FAA) NextGen plan, which will manage the increasingly congested airspace in the coming decades. While the benefits of ADS-B are widely known, its lack of security measures and its vulnerability to cyberattacks such as jamming and spoofing is a great concern for flight safety experts. In this paper, we first summarize the cyberattacks and challenges related to ADS-B’s vulnerabilities. Thereafter, we present theoretical and practical methods for implementing an Internet of Things (IoT)-based system as a possible additional safety layer to mitigate the presented cyber-vulnerabilities. Finally, a set of simulations and field experiments is presented to test the expected performance of the suggested IoT flight safety system. We conjecture that the presented system can be implemented in a wide range of civilian airplanes, leading to an improvement in flight safety in cases of cyberattacks or the absence of reliable ADS-B communication.

## 1. Introduction

Many states around the world have assessed and implemented ADS-B systems for airport surface or airspace surveillance [1]. ADS-B technology relies on the navigation system installed in the aircraft such as a Global Navigation Satellite System (GNSS) receiver to determine the position of the aircraft. The system integrates additional data and transmits the information on radio-based communication. Using this system allows air traffic controllers and aircraft to obtain accurate information about the aircraft’s locations and flight paths, which, in turn, allows for safer operations, more direct flight paths, and cost savings for operators [2,3]. To this end, understanding the ADS-B technology is necessary.

### 1.1. Related Works and Motivation

The ADS-B technology’s key challenges are related to the dependency on the GNSS as its primary positioning source, as well as the “fire to air” protocol based on Mode S extended squitters. The Mode S transponder is the basic protocol that supports the detailed communication between the aircraft transponder and the ground interrogator pair [1,4]. The ADS-B system used in commercial air traffic does not specify a mechanism to ensure trust protocol messages are authentic, nonreplayed, or adhere to other security properties. There are several technological mitigations, but their efficiency is yet to be proven. The authors of this paper [5] surveyed the theoretical and practical applications for the reader who wants to inquire further.

ADS-B can be used for several purposes and provides many benefits to both pilots and air traffic control. The main benefit, however, is the ability to visualize traffic information surrounding the aircraft, including the altitude, heading, and speed. This information allows improving air traffic conflict detection and resolution [1].

The newest aircraft such as the Boeing 787 have advanced flight decks that leverage state-of-the-art technology to improve their operational capabilities. The flight deck enables improved safety and reliability and provides a platform that can grow to support future air transport initiatives, such as ADS-B. Integrated surveillance systems provide reliable weather radar, transponder, Traffic Collision Avoidance System (TCAS), and ground proximity functionalities [6,7]. However, the number of works related to ADS-B security is steadily increasing as a result of the increasing use of the ADS-B system in the U.S. and other airspaces [8]. Modern aircraft experience a loss of position as a result of GPS disruption [9,10]. While the existing works touched on the need for security and offered insights into various aspects, this paper sought to present a novel IoT technology-based solution that will be an additional layer to protect aircraft from and mitigate cyberattacks. In this section, we cover the relevant background, emphasizing important systems and how they fit together.

### 1.2. Flight Safety

**Figure 1:**
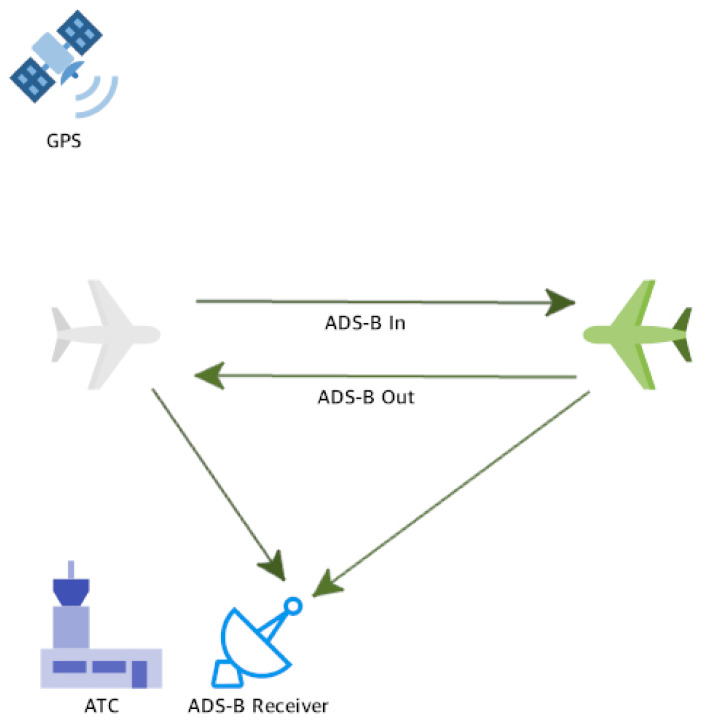
Aircraft use the GPS to determine their location and velocity and then transmit an ADS-B message containing these data using the ADS-B Out subsystem. ATCs use the ADS-B In subsystem to collect ADS-B messages from all aircraft in the area and then display a visual map containing these aircraft. There are aircraft equipped with the ADS-B In subsystem in addition to the ADS-B Out subsystem.

The ADS-B system uses an alternative way of displaying aircraft traffic compared to the use of the commonly used traditional radar. ADS-B messages are built from information collected from the aircraft system, as well as from the GPS mounted on the aircraft [5]. Typically, when aircraft are airborne, the ADS-B Out subsystem transmits extended squitters (long Mode S messages) twice per second for airborne position and airborne velocity [11]. The ADS-B In subsystem, which is located at a ground station or installed on the aircraft (ADS-B In for aircraft), receives and processes these messages and builds a map of the nearby aircraft that it detects (Figure 1). ADS-B messages may include additional information such as an urgency code entered by the pilot.

In addition to the ADS-B system, there is another system related to flight safety mounted on aircraft. The Traffic Collision Avoidance System (TCAS) is a safety system designed to prevent or reduce the severity of a collision between aircraft [1]. The interrogating TCAS receives the target aircraft altitude, and from the measured time delays, it computes the slant range to the target. Since the system uses a time delay, it has to use an accurate clock to calculate the Doppler shift effect. The system calculates the time when the two aircraft will be at the minimum distance from each other, including the height difference if any, and issues an alert to the pilots. Advanced TCASs have additional features such as coordinated actions for the pilots to climb or descend to maintain safe separation [12].

### 1.3. Flight Safety Vulnerabilities

**Figure 2:**
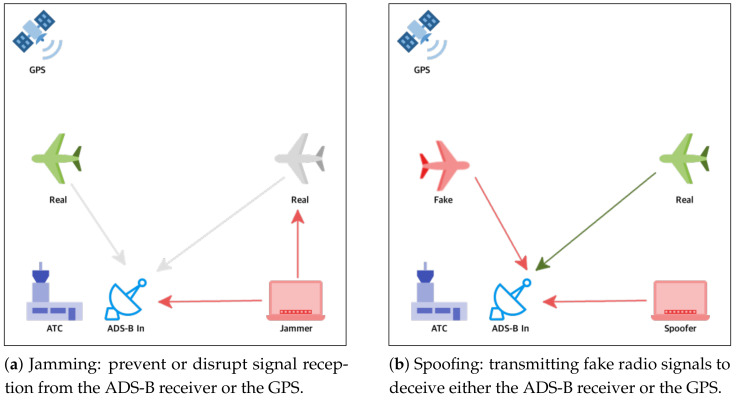
Jamming and spoofing cyberattacks on flight safety.

ADS-B consists of several other systems to collect the data for the ADS-B Out messages. It includes the GPS to determine the aircraft’s coordinates, a barometric altimeter for the aircraft’s altitude, and a transponder to send the messages. Each of these components has additional vulnerabilities to cyberattacks. However, this paper focused on the ADS-B system and the GPS being resilient on radio wave communication, while being vulnerable to cyberattacks such as jamming and spoofing (Figure 2), on a commercial aircraft.

### 1.4. Jamming

Jamming is the act of intentionally directing electromagnetic energy toward an RF-based receiver system to prevent or disrupt signal reception [11]. GNSS jammers broadcast their interference signal in the frequency band used for satellite navigation. A jamming attack can be categorized as a denial-of-service: the GNSS is still available, but its broadcast signals are exceeded by the jammer’s power. Interference as a result of another communication can be a reason for the denial of the GNSS services in a geographical location, but jamming refers to the intentional interference with the GNSS services in some locations [13]. Jamming in ADS-B is similar to GPS jamming. In particular, the jamming (Figure 2a) is targeted at the ADS-B In packets that are already in the air. The attack is performed by transmitting sufficiently high-power radio signals at a 1090 MHz frequency. This radio noise disables ground station ATCs or aircraft equipped with ADS-B In from reading and processing ADS-B messages.

### 1.5. Spoofing

Spoofing of an RF-based receiver refers to a deliberate and intentional transmission of fake or false radio signals to fool a system receiver into providing incorrect information [13]. Unlike jamming, the main intention behind a spoofing attack is to secretly force a GNSS receiver to track down the fake GNSS signals with the main objective of providing or, at least, inducing an incorrect navigation solution. ADS-B spoofing attacks are intended to create a false representation of a fake aircraft by transmitting fake ADS-B messages (Figure 2b). Since there is no authentication or other protection measures, it is easy to record real ADS-B messages using an ADS-B receiver and rebroadcast them later in a specific area or at a different time. Such an attack can be easily carried out using inexpensive and simple technological means such as Software-Defined Radio (SDR) and the GNURadio application and using widely available manuals on the Internet [4]. This type of attack is characterized by the presence of a fake aircraft flying in a collision path or by flooding a particular ATC airspace with fake aircraft [11].

### 1.6. Our Contribution

This paper presents a LoRa-based solution as an additional layer of protection against cyberattacks on commercial aviation safety. The proposed solution enables the mitigation of cyberattacks in both systems: ADS-B and GNSS. It was implemented as an IoT and sensor network system and tested in a simulation and field experiments. Preliminary results suggested that the concept can also be utilized as an improved sense and avoid system. To the best of our knowledge, this is the first research work to suggest the use of the LoRa 2.4 GHz protocol as a platform for ranging and ad hoc communication for civilian aviation. The presented concept can be implemented as an after-market safety product as it uses globally unlicensed ISM RF communication transceivers.

This paper is organized as follows: Section 2 introduces the problem of interest. Section 3 presents the flight safety framework including the protocols and technology used in the suggested solution. Section 4 covers the experiments performed using both the simulator and field experiments, and finally, Section 5 concludes this paper and discusses a few possible future work directions.

## 2. Problem of Interest

Various studies have been published on cyberattacks on the ADS-B system commonly used in commercial aviation [5]. These studies reviewed and raised a variety of possible cyberattacks on the ADS-B system and highlighted the problems of this system’s ability to defend against those attacks.

Flight safety vulnerability is affected not only by cyberattacks, but also by environmental conditions, malicious attacks, or even military experiments. In 2017, the FAA commissioned a board to look into the effects of intentional GPS interference on civilian aircraft [9]. Its report found that the number of military GPS tests had almost tripled from 2012 to 2017, while, unsurprisingly, GPS jamming was also on the rise.

Another flight safety incident report described how a passenger aircraft flew off course as a result of a GPS jamming attack and almost crashed into a mountain [10]. According to the report, two previous flights had advised that their GPS signals were interrupted, but then returned to working properly. The passenger aircraft reported a GPS problem as well and, shortly after, reported back that the problem was resolved. The aircraft began to descend before landing and was, therefore, transferred to a local airport control tower. Shortly thereafter, the ATC noticed that the plane had deviated from its path and approached a high-altitude mountain. Fortunately, the ATC contacted the local airport control tower and diverted the aircraft back to a safe trajectory.

Moreover, the FAA has begun outlining a rule that requires every Unmanned Aircraft (UA) operator to own a digital license called a Remote ID [14]. In its most basic form, remote identification is a kind of “digital license plate” for UAs. Although this rule is for UA operations, it highlights the need to address aviation safety and security issues in the airspace system.

## 3. Flight Safety Framework

In this section, we lay the foundations for our proposal that we present at the end of this section. We used devices and methods from the IoT world and describe solutions that were be based on them. We start with the description of the WSN and IoT and end with an expansion on the LoRa chip that was part of our solution.

### 3.1. Wireless Sensor Networks

Wireless Sensor Networks (WSNs) are becoming increasingly attractive in a variety of application areas, including industrial automation, security, weather analysis, and a broad range of military scenarios. WSNs are dense wireless networks of sensor nodes that collect and disseminate data [15]. Sensor nodes are small low-power devices severely constrained by their computation, communication, and storage capabilities, usually for economic reasons. They may sense around themselves, communicate over wireless channels at short ranges, and frequently, go into sleep mode to save power. A sensor node typically contains a power unit, a sensing unit, a processing unit, a storage unit, and a wireless transceiver. WSN platforms can be used with good results in a broad array of IoT applications.

### 3.2. Internet of Things

The IoT has become an emerging topic over the last few years. It can be described as the connection of devices or “things” across the Internet to deliver an assigned function [16]. This mainly comes in the form of devices sending or receiving data. Furthermore, GPS receivers enhance IoT devices to monitor and control their location and share this with other devices nearby.

### 3.3. LoRa Technology

Several technologies based on the IoT are Radio-frequency Identification (RFID), Near-Field Communication (NFC), and WSNs. These technologies are most often characterized by short-range and low-power communication capabilities that limit their coverage areas to a small range [16]. IoT-based technologies are usually designed for applications that broadcast sporadic short data such as beacons. However, multiple devices in the same application that transmit to the same ground station may become a bottleneck of the application.

In recent years, we have witnessed a rapid growth in the new technology referred to as Low-Power Wide-Area Network (LPWAN). This technology is characterized by long-range radio communication and utilizes the star network topology such that the data are transmitted from one device to another device or directly to a gateway and finally reach the Internet [16]. Another important feature of this new technology is the ability to place devices in a harsh environment where there is no other type of radio communication. LPWAN technology is operated in several different bands and frequencies depending on a country’s regulations. This technology can be broadly divided into two categories depending on the type of modulation: mainly Ultra-Narrow Band (UNB) or wideband, wideband allowing multiple devices to communicate on one channel.

Long-Range (LoRa) radio communication is based on a modulation called Chirp Spread Spectrum (CSS). This modulation uses its entire allocated bandwidth to broadcast a signal as chirp pulses to encode information [17]. Since it relies on a chirp pulse technique, it does not require the use of an accurate and stable internal clock, as in other transmission modulations, which are more expensive. This technique enables a variable data rate, thus providing the possibility to trade between efficiency of coverage and energy consumption while keeping a constant bandwidth. However, since this technology uses unlicensed bands, it must share radio communication with other devices using the same frequency [16]. Thus, it requires some regulation to ensure fair usage of the channel; hence, LoRa uses a duty-cycled transmission of the range from 0.1% up to 10%, depending on the sub-band [16].

### 3.4. LoRa 2.4 GHz Band

**Figure 3:**
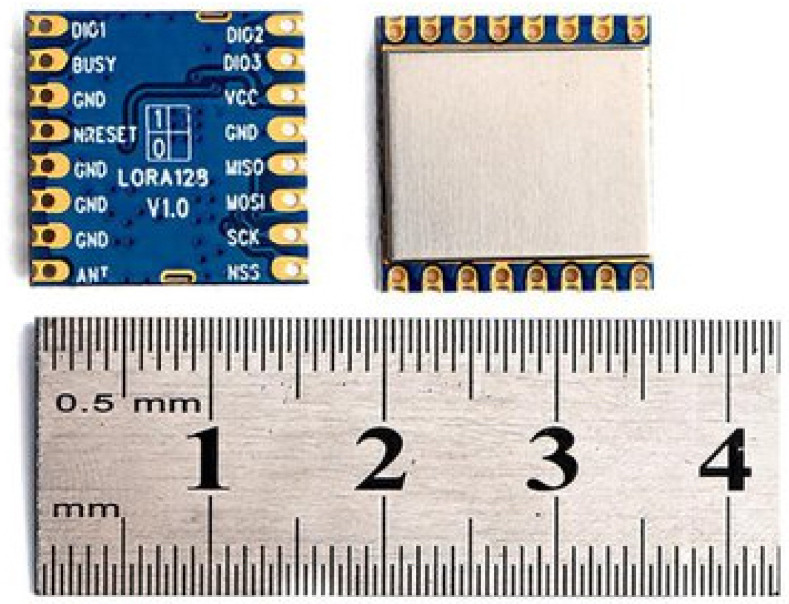
The LoRa 2.4 GHz module.

A new LoRa chip is available on the market, offering the 2.4 GHz ISM band with bandwidths up to 1.6 MHz and having a built-in Time-of-Flight (ToF) ranging engine [17]. Transmitting a message using this new chip is similar to transmitting a message with the previously described chip, but with higher data rates due to the higher bandwidth and frequency. The ToF ranging functionality is based on the measurement of a round-trip ToF between a pair of these new LoRa 2.4 GHz transceivers (Figure 3). This process is based on the following sequence. One module assumes the role of a master and initiates a ranging request message toward a receiver that has been configured as a slave. The master starts an internal timer once the ranging request message has been sent. Both the master and slave modules must be configured the same. The slave module that receives the ranging request message synchronizes itself with the incoming signal and sends the synchronized ranging response back to the master. The master then deduces the round-trip ToF from the time elapsed and calculates the ranging results between the two modules. Calibration and correction need to be performed on the ranging result to obtain a precise and accurate range measurement.

The ranging protocol between the two modules is based on data sent from the master to the slave without revealing the master’s information other than the request itself. Even if an adversary is able to respond to a ranging request, it will not be able to derive information other than the master’s ID. However, this can be resolved by using anonymous ranging requests.

### 3.5. Setup Implementation

**Figure 4:**
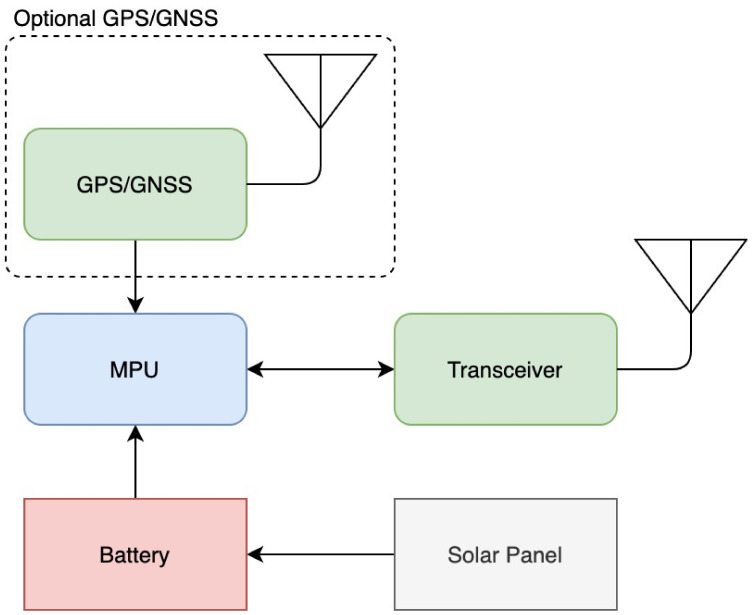
The node containing a microprocessor, a LoRa 2.4 GHz transceiver, and an optional GPS.

The basic sensor device (“node”) was built from a microprocessor, a radio transceiver, an optional GPS, a battery, and a solar panel for charging the batteries (Figure 4). Each node had the capability of sending data such as its location, neighbors, movements, and health to a nearby Ground Station (GS). The GS was a gateway application connected to the cloud in some way, and its function was to collect the nodes’ data and upload them to a cloud database for later use and analysis. On the other hand, using a UI system connected to the GS via the IoT, messages could be transmitted to the various nodes in the network. Since the transmission of such a node was limited to some range, a node could be operated as a relay and retransmit other nodes’ messages, allowing two unreachable nodes to communicate with each other. Many kinds of radio transceivers modules could be used by a node. However, a transceiver was required to communicate over a long distance while maintaining low energy to allow the node to operate over time. We proposed two setups that utilized the node device, and we next describe possible solutions to the related problems.

### 3.6. Wireless Sensor Network Protocol

**Figure 5:**
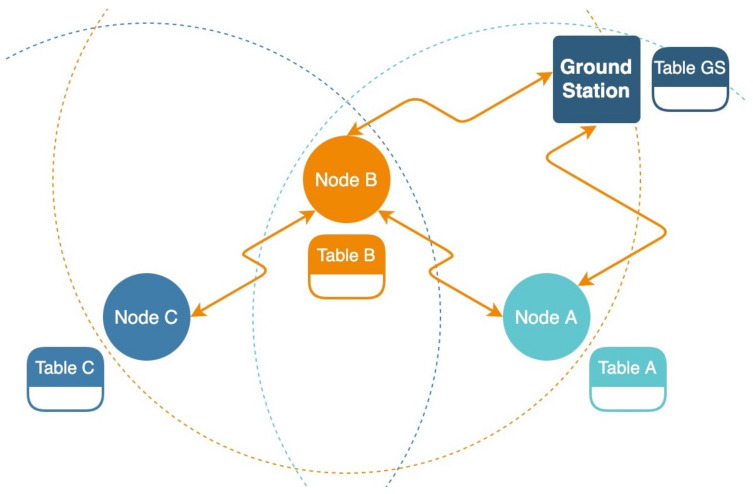
The wireless sensor network protocol diagram. Nodes A and B exchange information directly. Nodes A and C exchange information through Node B. The dotted rings represent the node’s transmit and receive ranges.

The wireless sensor network protocol between a node and a GS was as follows. Each node could send and receive messages using its integrated radio transceiver from or to any other node that exists in the network with its unique ID. Furthermore, each node contained a routing table of its neighboring nodes. Occasionally, a node would perform a health check with its neighbors and update its routing table. A message could be transmitted to a node directly if the receiver node was a neighbor of the origin node or through other nodes, which would act as relays and retransmit the message to their neighbors while ensuring that it was not sent back to the origin node (Figure 5). Each node contained a distance table in addition to the routing table. The distance table was constructed using the ranging feature of the radio device. Occasionally, a node would send a message to its neighbors to perform a ranging request. The distance table was published via a message to its neighbors to construct an entire network distance table. Using the distance table of its neighbors, a node could calculate its relative position using the following algorithm: If a node knew its distance from another node, then its position was known within a sphere. If there were two nodes to provide the distance, then the position was known within a ring of the two spheres. Together with a third node’s distance, its position was known on the three-sphere intersection. This intersection provided two possible points, but only one was on the Earth. Further, using a sensor to measure the altitude of the node, the position could be calculated precisely. Some nodes had their global position received by an integrated GPS sensor. Those nodes broadcast their GPS position as part of their beacon. Other nodes that did not have the integrated GPS sensor calculated their exact location using their distance table and their neighbors’ GPS location.

### 3.7. Sense and Avoid Protocol

**Figure 6:**
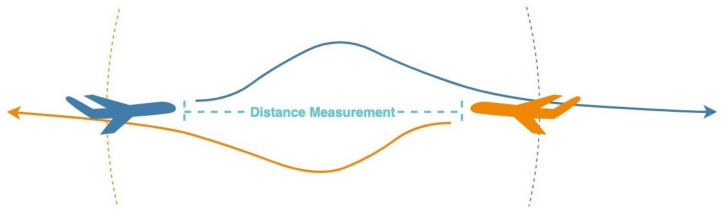
The node presents the measured distance between two airplanes. Further experiments showed the results of the measuring distances over 80 km.

The sense and avoid protocol functioned similarly to the TCAS presented in the Introduction, but it was implemented using the nodes described earlier with the following modification. A node was connected to an external power source and operated all the time. We assumed the node was integrated into an aircraft or into a moving object that had a power supply. The node continuously checked if there was another node in the area by sending an appropriate message. If a node responded in the positive, both nodes began measuring the distance from each other. If the distance measurement (Figure 6) recorded a result lower than a constant, the node displayed the proper alert.

At the same time, when a node received a positive answer regarding the distance measurement and started the process of measuring the distance, it was blocked from other nodes’ distance measurement requests. In this system, we focused solely on the ability to detect two nodes approaching each other at a distance that posed a danger to the aircraft. While modern TCASs provide additional information to the pilots such as flying the aircraft at different altitudes to avoid an impact [18], this proposal was an additional layer of protection while the TCAS was under a cyberattack.

### 3.8. Message Authentication

Authentication in the context of WSNs is the process of examining whether messages transmitted on the network were created by legitimate nodes of the network or were transmitted by non-network imposters. Message authentication is essential for transmitting messages on wireless sensor networks to prevent messages from being read by someone else and not by the specified end-user or receiver or to prevent the transmission of fake messages. For a good overview of the benefits and limitations of different messaging authentication techniques used in WSNs and ways to mitigate and protect against cyberattacks in IoT systems, the reader is referred to [15].

## 4. Simulator and Field Experiment

**Figure 7:**
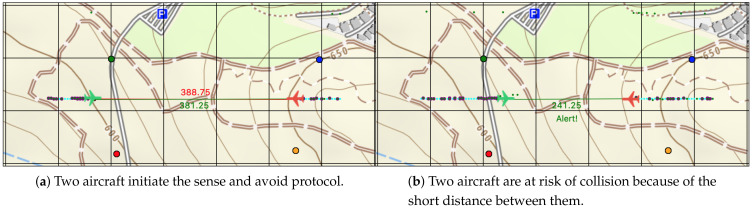
The large-scale simulator built for this study. These images display a simulation of the sense and avoid protocol for two aircraft.

Before moving to the field experiment and results, we present the simulator that was built for this study. The simulator was designed to analyze the proposed solutions in a large-scale environment that included multiple aircraft. The scenarios in this article pose a danger for aircraft in the real world. Furthermore, carrying out a cyberattack, in general, and on aircraft, in particular, is a violation of the law and even life-threatening. The simulator utilized the WSN and the sense and avoid protocols that were presented in the previous sections. These protocols were used in the field experiment as well, but in a small-scale environment. The simulator allowed building a WSN in a specific environment, adding multiple aircraft, experimenting with the sense and avoid scenarios (Figure 7), and simulating cyberattacks together with tools to verify and analyze the algorithms developed for the proposed solutions.

### 4.1. Wireless Sensor Network Experiments

**Figure 8:**
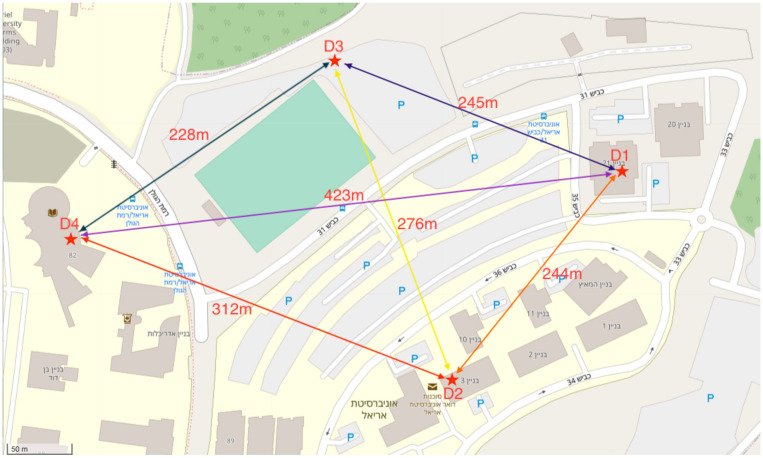
The layout of the nodes in the university area, as well as the distances between the nodes after the network started operating.

**Figure 9:**
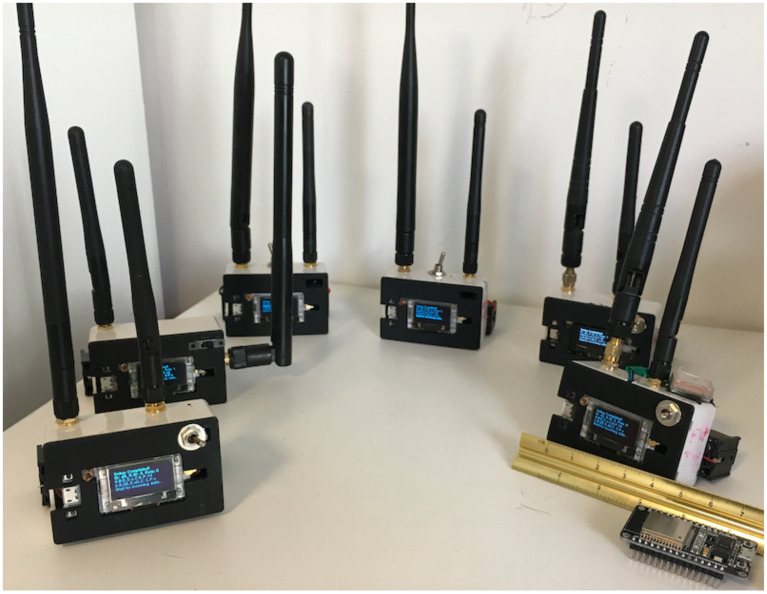
Several nodes were built to take part in the WSN field experiments.

This experiment was designed to test the feasibility of a WSN deployed in a given environment (Figure 8). Each node on the network contained a LoRa SX1280 2.4 GHz communication component, as well as an Arduino controller and a battery (Figure 9). Before the start of the experiment, the nodes that participated in the experiment were initialized and given a unique address. In addition, an initial adjustment of the communication component between both nodes in the network was made. This fine adjustment was required to increase the accuracy of the distance measurement. The adjustment was performed only once and was stored in the node memory. The nodes operated autonomously and managed their energy use. Occasionally, they conducted an interrogation of other nodes in the network, and if these nodes responded positively, they performed a distance measurement and stored the data in their memory. To preserve the network messages, a gateway node was setup, whose function was to receive network messages and store them in a cloud database.

At the end of the distance measurement, the node published its distance table to the network to allow other nodes verify their relative position with the use of the table. That is, when Node A reported that was at a certain distance from Node B, Node B performed a distance measurement and verified that it was indeed at the same distance as Node A had published. Similarly, Node B performed the same action with the rest of the nodes within its range. If a node found a different distance with respect to another node, it concluded that there was a problem and therefore temporarily suspended itself in the network.

The results showed that the distance table (Table 1) published by the nodes was similar to the distance table measured with the GPS (Table 2) within ±10%. The distance between the aircraft flying in a controlled airspace is usually several kilometers; therefore, we compared the accuracy level of the LoRa SX1280 2.4 GHz for longer distances compared to the GPS using weather balloons.

**Figure 10:**
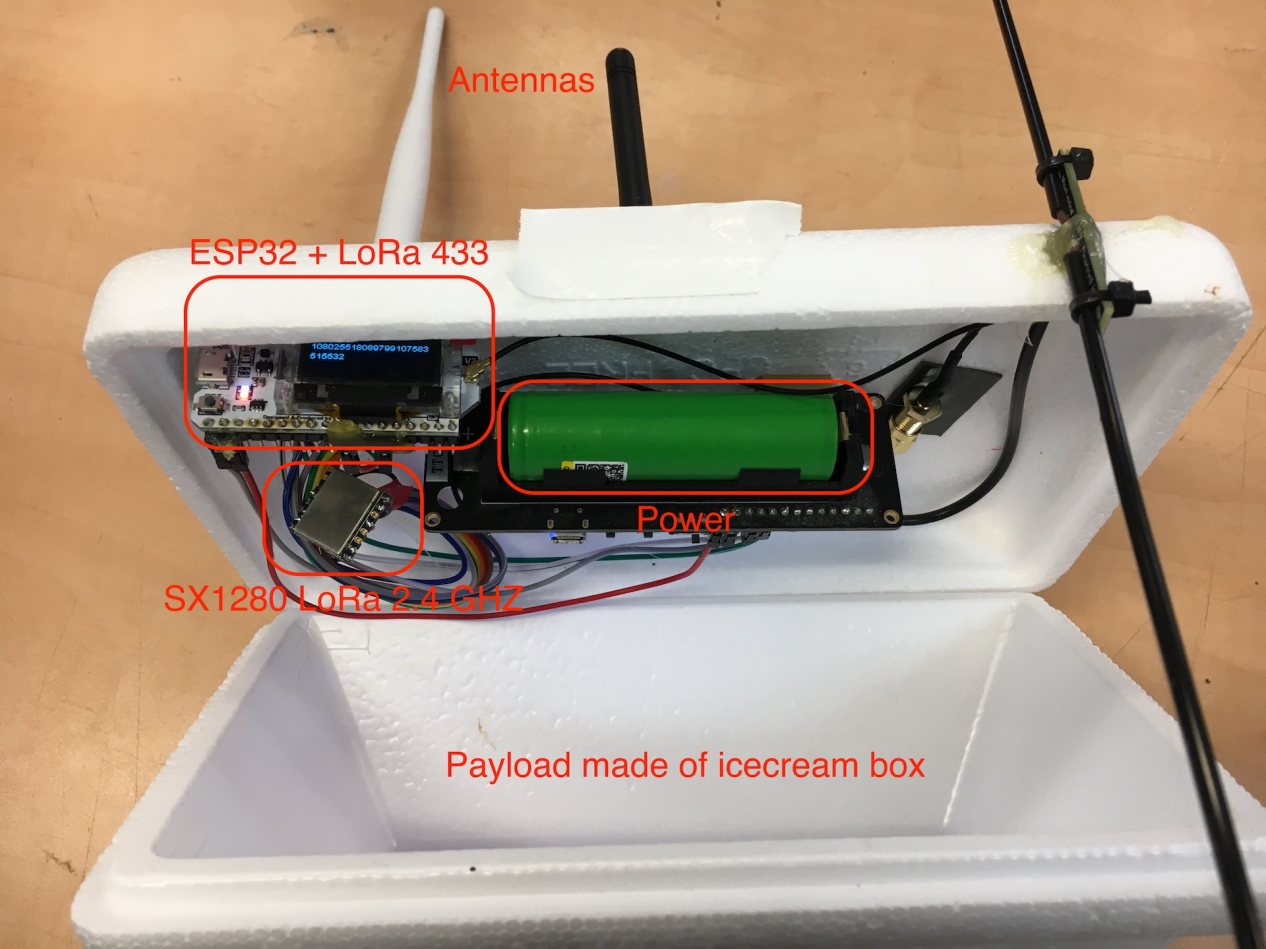
The payload for the long-distance ranging experiment included the LoRa SX1280 2.4 GHz and the LoRa 433 MHz for backup.

The distance measurements performed using this component and during the flight of the balloon (Figure 10) showed that they matched the GPS data obtained simultaneously with the beacon.

Table 3 shows a summary of the GPS data with the distance measurement data. The results showed that at distances exceeding 80 km, the error was up to ±10%. At the same time, the measurement results showed that as the distance increased, so did the level of accuracy. However, an accuracy level of ±10% is not sufficient for aircraft moving at distances of tens of kilometers, and therefore, more work is required to increase the accuracy level. Nevertheless, this experiment proved that it was feasible to use the setup in aircraft flying distances of tens of kilometers.

Performing a distance measurement between two nodes requires the use of the radio component. Thus, performing a distance measurement between two nodes prevents them from receiving messages from other nodes on the network. Therefore, the nodes aimed at measuring the distance at different times and as needed. That way, it was possible to facilitate network traffic and reduce the number of instances where nodes were not attentive to the network messages.

### 4.2. Finding Relative Position Experiments

We added a node with a new unique address to show the feasibility of an entity finding its relative position using a WSN. The new node moved in the network’s geographical area and performed an interrogation of which network nodes it could perform the distance measurements. After having received a positive response, it started performing the distance measurements with the responding nodes. At the end of the process, the node located its relative position according to the distances that it measured.

**Figure 11:**
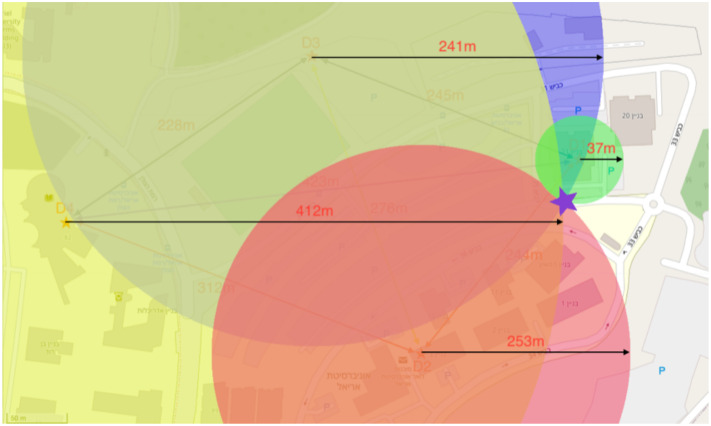
The node found its relative position using the wireless sensor network protocol.

Table 4 shows the distances obtained by performing the distance measurements against the other nodes in the network. Figure 11 shows the node’s location obtained by calculating the relative position on a map.

### 4.3. Sense and Avoid Experiments

In this experiment, we used two nodes representing two aircraft flying towards each other. The devices interrogated each other to continuously determine the distance between them. A third node was used as the GS and stored the data in the cloud database. Initially, the two nodes were placed at a distance of 200 m from each other. Following the initial distance measurement, the nodes moved towards each other and continued to measure their distance from each other. This experiment confirmed that distance measurements could be obtained for the sense and avoid system along with an alert message about a possible collision if the aircraft continued to move on the same path. However, the sampling time included a request time for sampling, and storing the data required about 2 s. At this time, the device was locked for distance measurement only.

### 4.4. Doppler Effect

When transmitters or receivers move relative to each other, the apparent frequency shift in the received signal is known as the Doppler effect. Transmission through LoRa was found to be immune to the Doppler effect. It has been found that this immunity allows the use of the LoRa modulation in satellite radio communications on routes over 500 km without the limitations related to the Doppler effect [19]. At the same time, we wanted to use the Doppler effect to estimate the relative position of the nodes. In a study conducted to investigate the Doppler effect on the LoRa components [20], it appeared that that the most affected transmission from the Doppler effect was in Spread Factor (SF) 12. Therefore, the researchers investigated the Doppler effect using vehicles moving at different speeds between 50 km/h and 80 km/h while transmitting a 50 bit message during a transmission time of more than 2 s.

The researchers noted that although LoRa was immune to the Doppler effect, it likely captured frequency anomalies due to the Doppler effect and predicted the speed of the moving node.

With this proposition, we could estimate the approaching or receding of two radio transmitters relative to each another. The Doppler effect would indicate whether two aircraft were moving away from or closer to each other. If they came closer, then using the Doppler effect, we could estimate the distance between the two aircraft and even verify the reliability of the aircraft. Aircraft flying in space and transmitting a radio signal cause the Doppler effect depending on their direction. Assuming that an aircraft reported movement in a certain direction, but the measurement of the Doppler effect indicated that it was not moving or even moving in the opposite direction, we could determine that it was an fake aircraft. If we added the Doppler effect as an additional layer of protection to the algorithms that we described in the previous sections, then it would be possible to check whether an aircraft’s responses to the WSN matched the Doppler effect. Otherwise, the system would report the possibility of a cyberattack such as an impersonator.

## 5. Conclusions and Future Work

In this study, we utilized IoT methodologies in order to address the cyber-vulnerabilities affecting public flight safety. After reviewing the literature, it appeared that the solutions presented so far tried to add layers of security such as encryption or authentication to the existing ADS-B system.

This paper described IoT components implemented as autonomous devices as part of a WSN that could be deployed in any area and provide an additional layer of protection for aircraft flying in that airspace. The experiment results showed that this system could detect and mitigate a cyberattack on the aircraft ADS-B and GPS. Furthermore, it allowed an aircraft to find its relative position and even its global position. In addition, the same installation could be used as a standalone sense and avoid system based on similar sensors that could alert about an expected collision between two aircraft. The advantage of such a solution was the use of an external system that was independent of legacy systems and therefore simpler to implement and install on existing aircraft. Furthermore, the presented framework was inexpensive, had low energy consumption, and was capable of transmitting short data packets over long distances. The proposed system could also be deployed as a ground-based ad hoc solution in areas where cyberattacks are suspected.

As future work, we would like to implement a sense and avoid system for a swarm of UAVs. Moreover, we plan to generalize existing remote control open-source platforms such as ExpressLRS [21] (based on the LoRa SX1280 transceivers) to allow one-to-many and mesh networks between drones and ground sensors.

## Figures and Tables

**Table 1:** Distance measurement results using LoRa SX1280 2.4 GHz.

Device	D1	D2	D3	D4
D1	-	250.2	252.8	416.2
D2	248.4	-	230.2	344.8
D3	242.9	242.9	-	219.2
D4	431.8	288.7	312.0	-

**Table 2:** Distance measurement results using GPS.

Device	D1	D2	D3	D4
D1	-	244.0	245.0	423.0
D2	244.0	-	276.0	312.0
D3	245.0	276.0	-	228.0
D4	423.0	312.0	228.0	-

**Table 3:** The LoRa SX1280 2.4 GHz distance measurement compared to the GPS using a long-range weather balloon.

GPS	LoRa SX1280	Difference	Time Shift	Accuracy
(Meters)	(Meters)	(Meters)	(Seconds)	
1780	1980	200	12	92.93%
2130	2404.6	274.6	29	91.26%
13,210	13,453.7	243.7	17	98.75%
21,530	21,698.8	168.8	11	99.64%
22,170	22,325.1	155.1	11	99.70%

**Table 4:** Node distance measurement results.

Device	D1	D2	D3	D4
A1	36.7	253.1	241.3.0	412.0

## Data Availability

The data presented in this study are available on request from the corresponding authors.

## Abbreviations

The following abbreviations are used in this manuscript:

ADS-B	Automatic Dependent Surveillance-Broadcast
ATC	Air Traffic Control
FAA	Federal Aviation Administration
GNSS	Global Navigation Satellite System
ISM	Industrial, Scientific, Medical
IoT	Internet of Things
RSSI	Received Signal Strength Indicator
SNR	Signal-to-Noise Ratio
TCAS	Traffic Collision Avoidance System
TIS-B	Traffic Information Services-Broadcast
ToF	Time-of-Flight
UAV	Unmanned Aerial Vehicle
WSN	Wireless Sensor Network
